# Optimal Allocation of Node Capacity in Cascade-Robustness Networks

**DOI:** 10.1371/journal.pone.0141360

**Published:** 2015-10-23

**Authors:** Zhen Chen, Jun Zhang, Wen-Bo Du, Oriol Lordan, Jiangjun Tang

**Affiliations:** 1 School of Electronic and Information Engineering, Beihang University, Beijing 100191, People’s Republic of China; 2 Beijing Key Laboratory for Network-based Cooperative Air Traffic Management, Beijing 100191, People’s Republic of China; 3 School of Engineering & IT, University of New South Wales at the Australian Defence Force Academy, Canberra, Australia; 4 Universitat Politècnica de Catalunya-BarcelonaTech, C/Colom no. 11, Terrassa 08222, Spain; Southwest University, CHINA

## Abstract

The robustness of large scale critical infrastructures, which can be modeled as complex networks, is of great significance. One of the most important means to enhance robustness is to optimize the allocation of resources. Traditional allocation of resources is mainly based on the topology information, which is neither realistic nor systematic. In this paper, we try to build a framework for searching for the most favorable pattern of node capacity allocation to reduce the vulnerability to cascading failures at a low cost. A nonlinear and multi-objective optimization model is proposed and tackled using a particle swarm optimization algorithm (PSO). It is found that the network becomes more robust and economical when less capacity is left on the heavily loaded nodes and the optimized network performs better resisting noise. Our work is helpful in designing a robust economical network.

## Introduction

Modern human societies very much depend on large scale critical infrastructures to deliver resources and services to consumers and businesses. These infrastructures are complex systems of structural and functional elements. Such systems work reliably in our everyday life. However, as some rare occurrences in the past have shown, they are still vulnerable to major outages, such as telecommunication outages [1], blackouts in power grids [2–4] and financial bankruptcy [5].

The theory of complex networks has emerged in recent years, and has proved to be a valid tool to describe, model and quantify complex systems in system robustness and cascading failures [6–16]. In these fields, two important issues are always focused on by most researchers: how to design man-made networks with higher robustness to cascading failures and how to reduce the cost to maintain the robustness of networks. High robustness and low cost seem to be contradictory, and it is difficult to achieve both in most cases. For instance, dense networks are more likely to be robust to cascading failures, but more edges always mean more resources and a higher cost. In short, building a robust network is very expensive.

Some models and strategies have been proposed to balance robustness and cost. Motter et al. [17] first introduced a homogeneous capacity-load relationship model which is widely used today. In this model, the capacity of a node is proportional to the initial load of the node which is based on the flow along the shortest hop path. Wang et al. [18] introduced a model in which the capacity of a vertex is assigned as a nonlinear monotonically increasing function of the load. It aims to enhance the robustness of the network with a lower cost via protecting the higher load vertices merely.

However, most previous works allocate the capacity resources simply according to network topology information, such as degree and betweenness. Kim et al. [19, 20] argued that these models are unrealistic due to the fact that empirical networks always show a nonlinear capacity-load relationship and heavily loaded nodes usually have relative less unoccupied capacity. Actually, the essence of resource allocation is an optimization problem, but the cascading failure process is hard to be described functionally, rendering traditional optimization methods powerless. It was not until very recently intelligent optimization algorithms, which have been proven to be valid for solving practical non-functional optimization problems, have been applied to network optimization [21–25]. Huang et al. [21] proposed a multi-objective simulated annealing algorithm to optimize the network topology for packet routing. Zhou et al. [22] introduced a memetic algorithm to optimize the structure of networks in order to enhance the robustness of scale-free networks against cascading failures. Fang et al. [23] applied the non-dominated sorting binary differential evolution algorithm to the power generation allocation of the existing buses in the 400kV French power transmission network. It turns out that optimization algorithms work well in optimizing the robustness of complex networks.

Motivated by their works, in this paper we formulate the problem of resource allocation within a large-scale, nonlinear and multi-objective optimization framework and construct an optimal model of allocating the capacity resources. To solve it, an effective algorithm named particle swarm optimization (PSO) is utilized. We investigate the cost-efficiency, capacity-load and vulnerability-cost relationships and the effect of noise.

## Materials and Methods

In this section, we describe (1) network and cascade model, (2) optimal model of capacity allocation, and (3) PSO algorithm based solution. For clarity, the terms “node” and “vertex” will be used interchangeably in this paper.

### Network and cascade model

To capture the heterogeneity of many real-world networks, the Barabási-Albert (BA) scale-free network [26] is adopted. The BA network is generated by starting from a small amount of *m*_0_ fully connected nodes. It increases by adding a new node at each time step. This new node is connected preferentially to *m*(*m* ≤ *m*_0_) old nodes in such a way that the probability of connecting to an existing node is proportional to the old node’s degree. The BA scale-free network exhibits a power-law distribution *P*(*k*) ∼ *k* ^−*γ*^. In the following, the networks are set to *N* = 500, *m*_0_ = 2 and *m* = 2.

In the cascade model, the node load is estimated through node betweenness if the traffic flow travels along the shortest-path. As in Refs. [17, 27] we adopt the data-transport model which uses node betweenness to denote the node load:

$$L_{i}=\sum_{s\neq i\neq t}\frac{\theta_{st}^{i}}{\theta_{st}}$$ (1)

where $\theta_{st}^{i}$ is the number of shortest paths between nodes *s* and *t* that run through node *i*, and *θ*_*st*_ denotes the total number of available shortest paths from *s* to *t*. The capacity of a node is the maximum load that the node can handle. Following Refs. [17, 18], the capacity of node *i* is assigned as

$$C_{i}=(1+\alpha_{i})L_{i}^{0}$$ (2)

Where $L_{i}^{0}$ is the initial load and *α*_*i*_(*α*_*i*_ ≥ 0) is the tolerance parameter.

The cascading failure is triggered by removing the highest-load node in the network resulting in the globally redistribution of the flow and node loads [17, 18]. If a surviving node is overloaded, i.e. *L*_*i*_ > *C*_*i*_, then it fails, leading to further load redistribution. This process continues recursively until no more nodes overload. Moreover, it is assumed that only the nodes in the giant component remain functional.

### Optimal model of capacity allocation

Methods of resource allocation are generally framed to reduce the vulnerability (enhance the robustness) of networks against cascading failures with a limited cost. The variables to be optimized are defined as the tolerance parameter vector $\vec{\alpha }$ (i.e. $\vec{\alpha }=[\alpha_{1},\alpha_{2},\ldots ,\alpha_{N}]\geq 0$, is a vector of network size *N*). Motter-Lai (ML) model [17] is the most basic and widely used model, in which *α*_*i*_ are the same values *c*(*c* > 0) for all nodes. Wang et al. [18] proposed a model in which $\alpha_{i}=c\Theta (\frac{L_{i}}{L_{\text{max}}}-\beta )$, where Θ(*x*) = 0(1) for *x* < 0(*x* > 0), is a two-valued function. The latter model performs better than the former one, so it is possible that a pattern of $\vec{\alpha }$ that yields lower vulnerability and cost can be found by means of a variation approach.

The vulnerability of a network is defined as

$$V(\vec{\alpha })=\frac{N^{'}(\vec{\alpha })}{N}$$ (3)

where $N^{'}(\vec{\alpha })$ is the number of failed vertices under the tolerance vector $\vec{\alpha }$ and *N* is the total number of vertices in the network. Obviously, the smaller *V* is, the more robust the network will be. The cost of whole network resources is defined, in accordance with previous works [18], as

$$E(\vec{\alpha })=\frac{1}{N}\sum_{i=1}^{N}\alpha_{i}=\frac{1}{N}\vec{\alpha }\cdot \vec{1}$$ (4)

where $\vec{1}$ is a column vector of ones. The larger the *E* is, the more resources the network costs.

The cascade-robustness network is a network with redundant vertex capacity. However, redundancy usually means extra cost. Robustness and cost are expected to be in opposition, requiring trade-offs. Therefore, we define the overall objective function $F(\vec{\alpha })$ of a network by aggregating vulnerability and cost through an adjusting parameter *ω*(0 < *ω* ≤ 1) balancing the importance of vulnerability and cost:

$$F(\vec{a})=\omega V(\vec{a})+(1-\omega )E(\vec{a})$$ (5)

When *ω* is small, we pay more attention to controlling the cost rather than reducing the vulnerability. On the contrary, when *ω* is large, we focus more on enhancing the network robustness and care less about the cost. It is easily found that the smaller the objective function is, the better the results are.

Maximum cost is always a constraint in real world projects, which are subject to a budget. Thus, it is necessary to set whole network maximum cost as a constraint. Since ML model is the fundamental model, without loss of generality, we set the whole network cost of the initial pattern of ML model, as the maximum cost.

Then the node capacity allocation optimization model can be formulated as

$$\begin{array}{l} \text{min}\;F(\vec{\alpha })\\ s.t.\;\left\{ \begin{array}{l} E(\vec{\alpha })\leq \lambda \\ E(\vec{\alpha })=\frac{1}{N}\vec{\alpha }\cdot \vec{1}\\ \vec{\alpha }\geq 0 \end{array}\right. \end{array}$$ (6)

The dimension of the variable $\vec{\alpha }$ is *N*, which represents the number of vertices in the network. $F(\vec{\alpha })$ is the objective function which is the linear combination of vulnerability and cost and is not an analytic expression. *λ* is the upper bound of the total node capacity cost. The value $\vec{\alpha }$ is a feasible solution if it satisfies all the constraints, otherwise it is an infeasible solution. One feasible solution is $\vec{\alpha }=[\lambda ,\lambda ,\ldots ,\lambda ]$, which means feasible solutions do exist.

### Particle swarm optimization based solution

Particle swarm optimization is an effective optimization algorithm proposed by Kennedy and Eberhart [28] which is inspired by the social behavior of swarms such as fish schooling or bird flocking [29]. In PSO, to search for the global optimum, a flock of particles move in a constrained parameter space, interacting with each other and updating their positions and velocities. Owing to its simplicity, effectiveness and low computational cost, PSO is widely used in solving practical optimization problems [30, 31] and there are a number of variations [32–36].

For our capacity allocation optimization problem with *N* variables and an objective function $F(\vec{\alpha })$, the PSO algorithm represents the potential solutions with a flock of particles. Each particle *i* has a position $\vec{x_{i}}=[x_{i1},\;x_{i2},\;\ldots x_{iN}]$ and a velocity $\vec{v_{i}}=[v_{i1},\;v_{i2},\;\ldots v_{iN}]$ in the *N*-dimensional space. The position $\vec{x}$ represents the tolerance parameter $\vec{\alpha }$ and the velocity $\vec{v}$ represents the variance of $\vec{\alpha }$. The goal is to find an optimal position $\vec{x}$ of any particle *i* that makes the objective function $F(\vec{\alpha })$ minimum. Initially the particles’ positions and velocities are generated randomly within the constraint. Then, at each iteration each particle updates its position and velocity according to the following equations [28]:

$$\vec{v_{i}}=\eta \cdot (\vec{v_{i}}+\vec{U}(0,c_{1})\cdot (\vec{p_{i}}-\vec{x_{i}})+\vec{U}(0,c_{2})\cdot (\vec{p_{n,i}}-\vec{x_{i}}))$$ (7)

$$\vec{x_{i}}=\vec{x_{i}}+\vec{v_{i}}$$ (8)

where $\eta =\frac{2}{\left|2-\varphi -\sqrt{\varphi^{2}-4\varphi }\right|}$, *φ* = *c*_1_ + *c*_2_ > 4. Here *p*_*i*_ is the best historical position found by *i*’s neighbors, *c*_1_ and *c*_2_ are the acceleration coefficients. $\vec{U}(a,b)$ is a random number drawn at each iteration from the uniform distribution [*a*, *b*]. Therefore, *c*_1_ and *c*_2_ balance the impacts of each particle’s own and its neighbors’ experiences, and *η* indicates the learning rate. Based on previous extensive analysis [37], we choose the appropriate setting as *c*_1_ = *c*_2_ = 2.05 and *η* = 0.7298. The number of particle is 50 and the number of iterations is 1000.

To handle the constraints, we follow Ref. [38]:

1. A volition function $G(\vec{\alpha })$ is assigned to make up the final fitness function with $F(\vec{\alpha })$. The volition function is defined as follows:

$$G(\vec{\alpha })=\left\{ \begin{array}{l} 0,\;if\;E(\vec{\alpha })\leq \lambda \\ E(\vec{\alpha })-\lambda ,\;otherwise \end{array}\right.$$ (9)

2) A better solution is chosen by the tournament selection operator. The following three criteria are satisfied during the selection operator:
  - when two feasible solutions are compared, the one with smaller value of $F(\vec{\alpha })$ is chosen;
  - when two infeasible solutions are compared, the one with smaller value of $G(\vec{\alpha })$ is chosen;
  - when one feasible and one infeasible solutions are compared, the infeasible solution is chosen only if it has a smaller value of $F(\vec{\alpha })$ and $G(\vec{\alpha })$ is less than a constant *ε*, otherwise the feasible solution is chosen.
3) The constant *ε* is used to limit the number of infeasible solutions. To keep the proportion of infeasible solutions a fixed proportion *l*, *ε* is defined as:

$$\varepsilon =\left\{ \begin{array}{l} 1.2\varepsilon ,\;\text{when the proportion of infeasible soloution}<l\\ \varepsilon ,\;\text{when the proportion of infeasible soloution}\;=l\\ 0.8\varepsilon ,\;\text{when the proportion of infeasible soloution}\;>l \end{array}\right.$$ (10)

It is worth noting that a handful of infeasible solutions do exist in the population. They are helpful due to the fact that sometimes the optimal solution is found in the boundary of the constraints.

## Results and Discussion

Solving the optimal model of allocation of resources using the PSO algorithm described in previous section, we analyze (1) the optimization performance of the network; (2) cost-efficiency, (3) capacity-load and (4) vulnerability-cost relationships; and (5) the effect of noise.

### Optimization performance of the network

**Fig 1:**
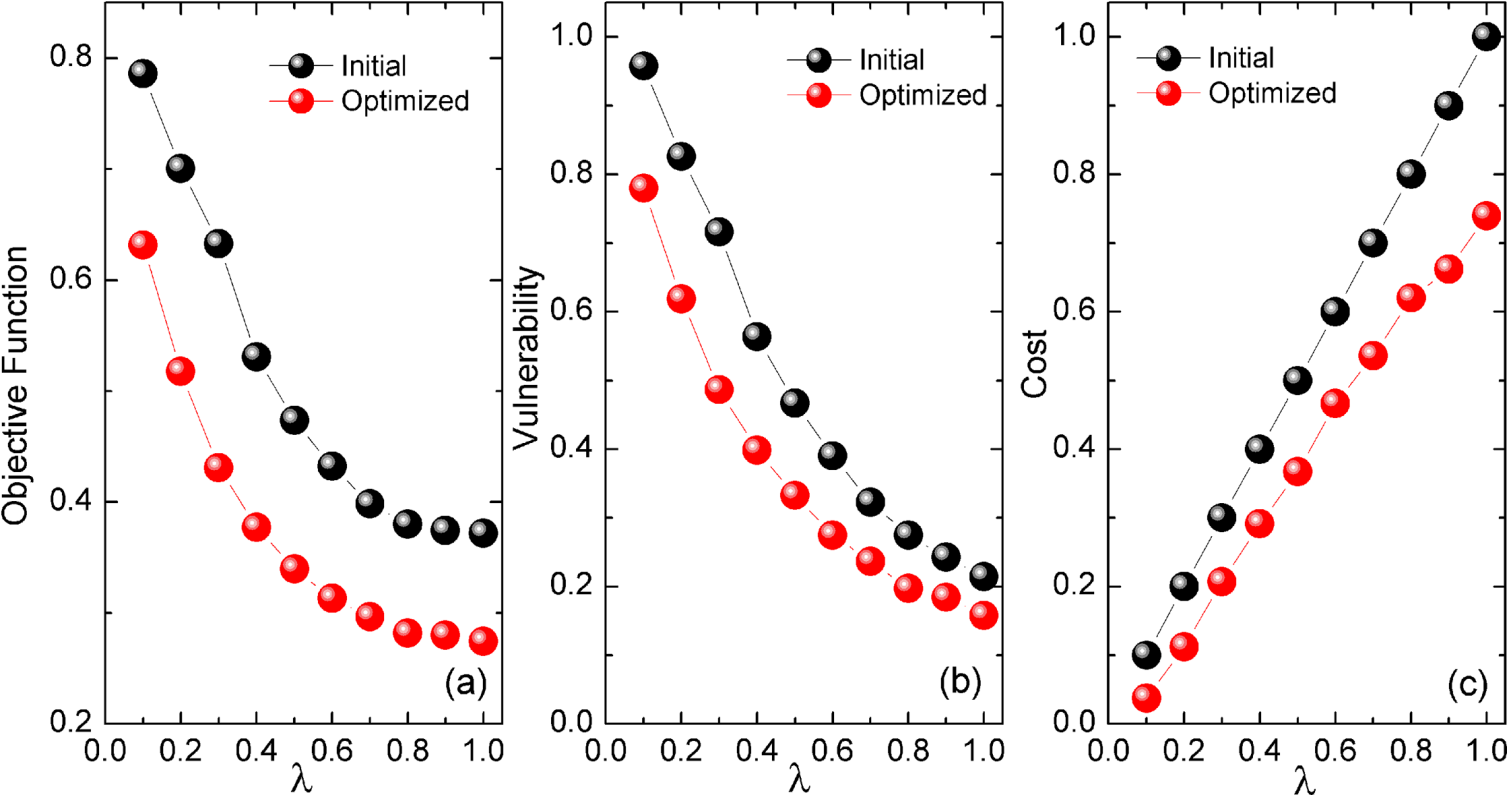
Performance of the optimized pattern and the initial model pattern under different maximum costs. (a) Objective function. (b) Vulnerability of the network. (c) Cost of the network. Each point is averaged from 20 different networks. The adjusting parameter is set to *ω* = 0.8. The algorithm runs for 1,000 iterations each time.

We start by analyzing the optimization performance on BA scale-free networks. The basic Motter-Lai model [17] is set as the initial model to compare with. As reducing the vulnerability is the main purpose of real network design, we set the adjusting parameter *ω* = 0.8. In our optimization model this is also a well-considered trade-off which is discussed in the part of vulnerability-cost relationship of the network. Fig 1A shows that the objective function is much smaller after optimization for all values of *λ*. The optimized capacity allocation pattern does perform better than the initial model pattern. To get a full scenario, Fig 1B and 1C show the vulnerability and cost of the network, respectively. The network’s vulnerability decreases monotonously as the maximum cost increases in both optimized and non-optimized situations, which is in agreement with the intuition that more redundancy results in lower vulnerability. The optimized networks are always less vulnerable than the initial ones. Regarding the cost, the optimized pattern’s cost is only approximately 70% of the initial pattern. Consequently, the optimized networks are more robust at a lower cost.

### Cost-efficiency relationship of the network

In order to further investigate the robustness and cost of the network, we set up a cost-efficiency indicator:

$$H(\vec{\alpha })=\frac{1-V(\vec{\alpha })}{E(\vec{\alpha })}$$ (11)

**Fig 2:**
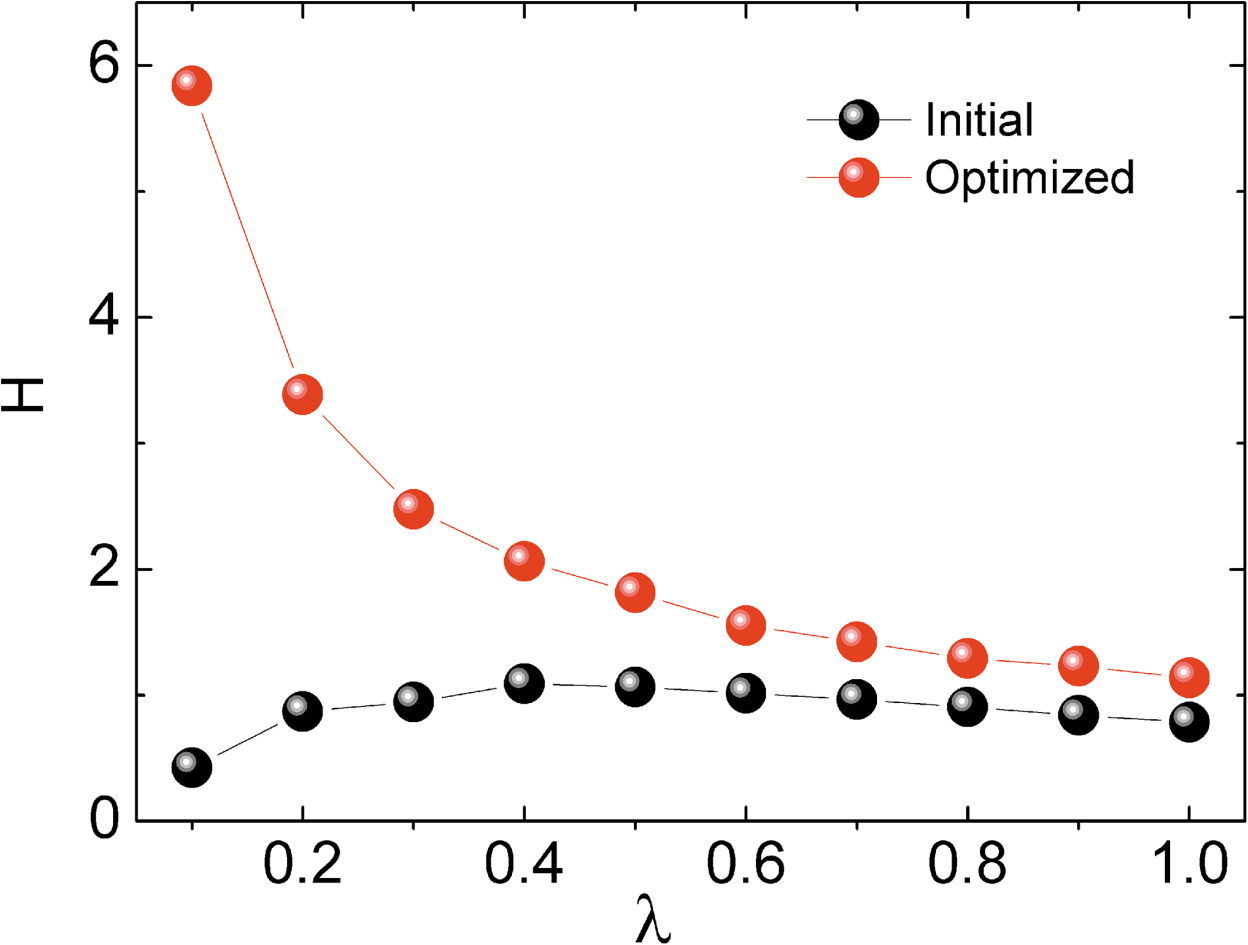
The cost-efficiency indicator *H* of different maximum costs. Each point is averaged from 20 different networks. The adjusting parameter is set to *ω* = 0.8. The algorithm runs for 1,000 iterations each time.

Here *H* represents the average robustness gain from a unit cost, which is akin to the marginal utility in economics. Fig 2 shows that the cost-efficiency of optimized pattern is higher than that of initial pattern, especially when the maximum cost *λ* is small. In general terms, if the resources are allocated in the optimized pattern, the unit cost can produce much more robustness benefit, and more nodes will be able to survive a cascading failure. Besides, as the *λ* increases, each homogenous unit cost contribution to the network’s robustness becomes smaller. This interesting observation is just like the law of diminishing marginal utility. It shows that it is not wise to invest too much extra cost in protecting facilities when designing a system.

### Capacity-load relationship of the network

**Fig 3:**
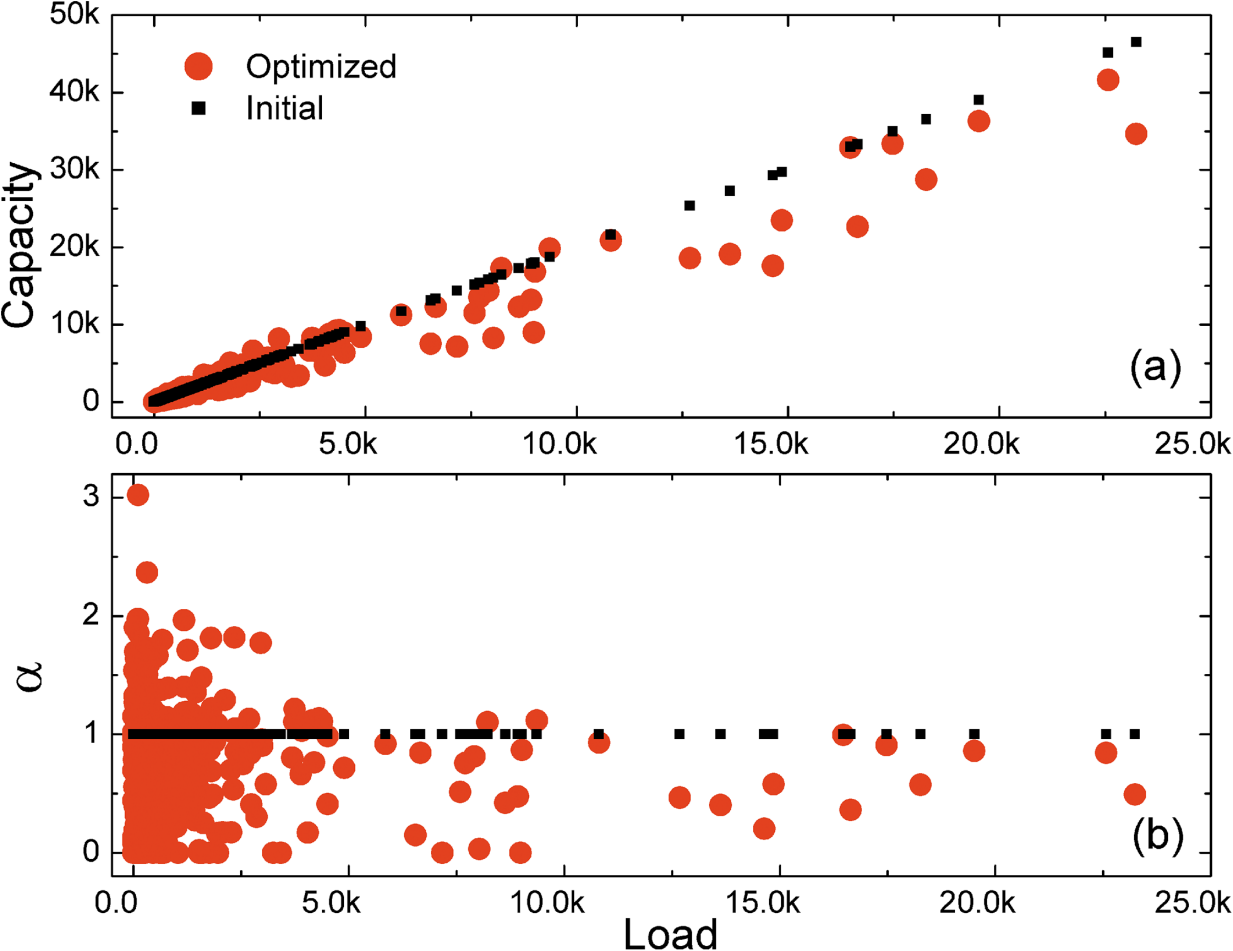
Capacity-load relationship of the network. (a) Node capacity as a function of node initial load. (b) Node tolerance parameter *α* as a function of node initial load. The adjusting parameter is set to *ω* = 0.8. The maximum cost *λ* = 1.0. The algorithm runs for 1,000 iterations each time.

Now we investigate the relationship between optimized node capacity and initial node load in the network reported in Fig 3. The capacity-load relationship in the initial model is linear and the tolerance parameter *α*_*i*_ is the same for all nodes. On the contrary, the relationship in the optimized model is nonlinear and *α*_*i*_ changes depending on the node. What is more, Fig 3 shows that nodes with large initial loads tend to have less unoccupied capacity in the optimized model. This interesting phenomenon is due to the fact that the flow fluctuations in heavily loaded nodes are relatively small when cascading failures occur and flow redistributes. Assigning excessive unoccupied capacity to heavily loaded nodes is actually a waste of resources and cannot reinforce the robustness of the network greatly. Assigning more resources to some lightly loaded but fragile nodes or simply taking out these redundancy capacities is a better alternative. It is worth to note that this finding is consistent with the empirical observations and results such as airports network, power grids and Internet router network [19, 20].

### Vulnerability-cost relationship of the network

**Fig 4:**
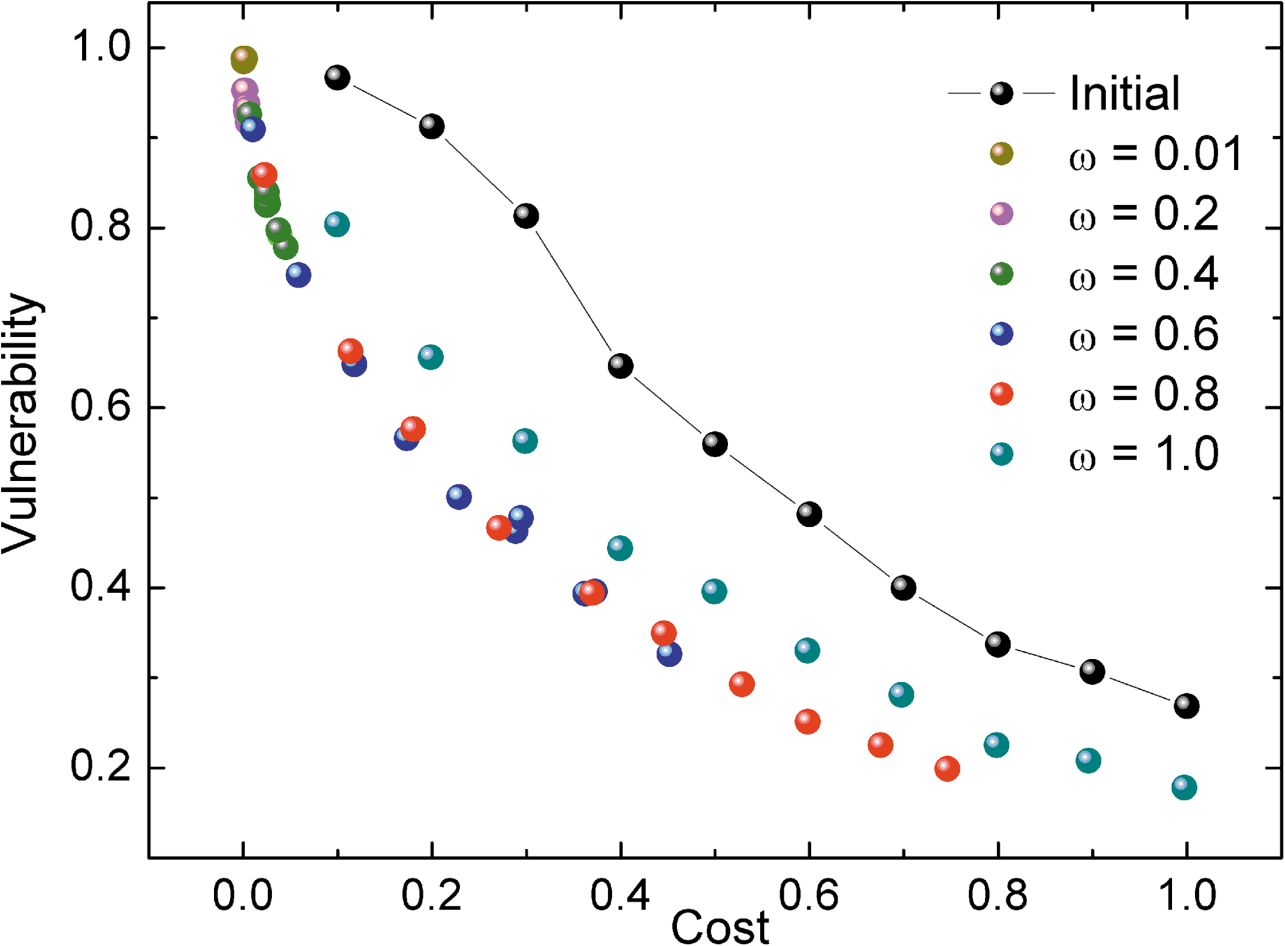
The Pareto frontiers of various values for parameter *ω*. Each point is averaged from 20 different networks. The algorithm runs for 1,000 iterations each time.

There is a critical parameter in the optimal model of allocation of resources, *ω*. It is a trade-off of the vulnerability and the cost in the objective function. Fig 4 shows the Pareto frontier of *ω* from 0.01 to (considering that the vulnerability of the network should be taken into account, *ω* cannot be equal to 0). As we can see, all the solutions of the initial model pattern are dominated by the solutions of the optimized pattern, which means that no matter what the adjusting parameter *ω* is, the results of the optimized model are always better. When *ω* is relatively small, the solutions mainly gather in the upper left corner. Networks that follow these node capacity allocation patterns possess a lower cost as well as a higher vulnerability. When *ω* is relative large, the solutions tend to distribute evenly. Networks following these allocation patterns are more robust against cascading failures at a relatively lower cost. Therefore, the parameter *ω* can be chosen in accordance to the actual demand. It should also be noted that the solutions for *ω* = 1 do not lie on the frontier, and they are dominated by other solutions, indicating that enhancing robustness at all costs is not a good solution. *ω* = 0.6 and *ω* = 0.8 are well-considered trade-offs of vulnerability and cost.

### Noise effect

**Fig 5:**
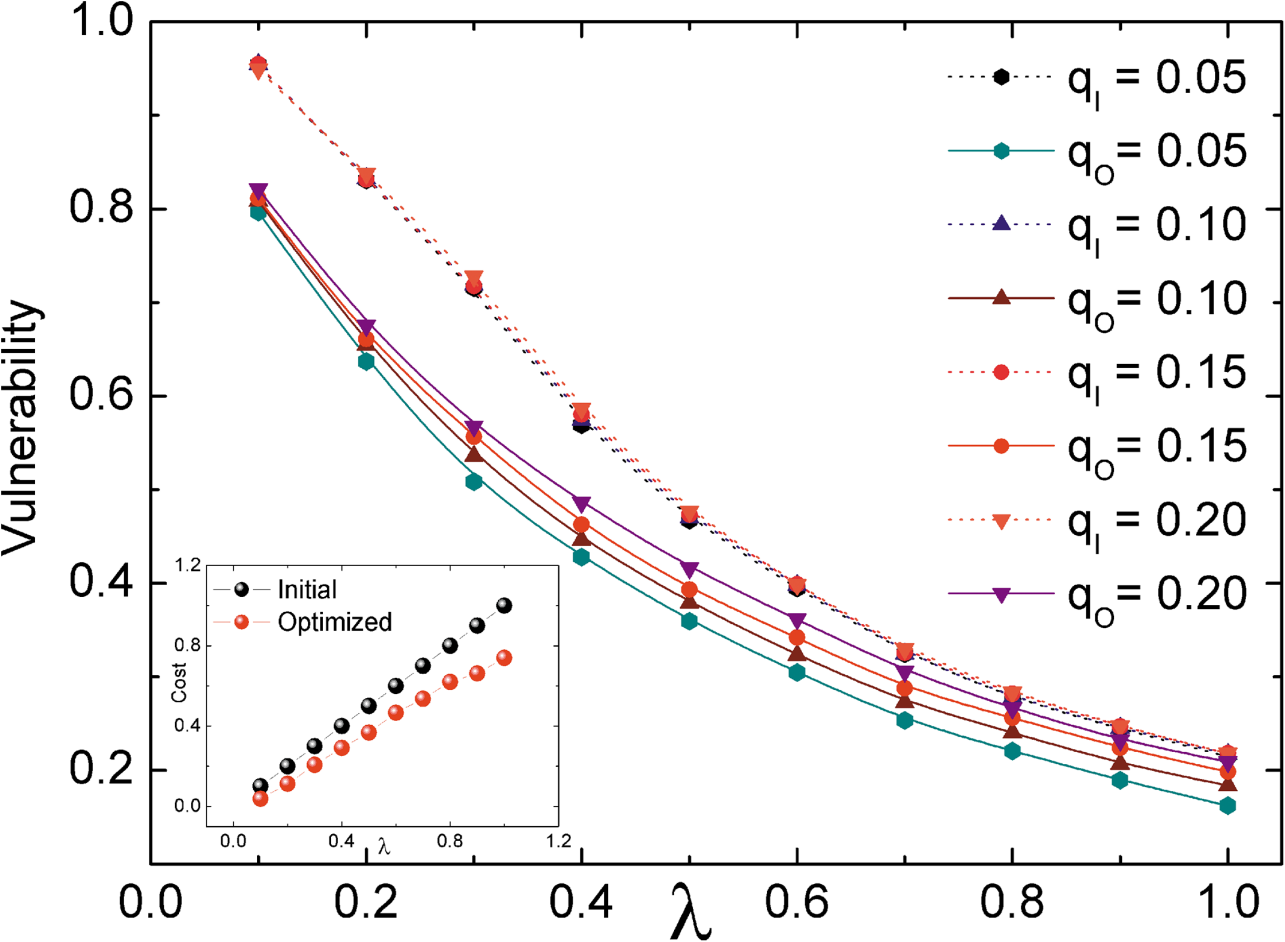
The effect of noise. The dot lines represent the initial networks and the solid lines represent the optimized networks. Each point is averaged from 20 different networks. The adjusting parameter is set to *ω* = 0.8.

In reality, nodes capacity is inevitably fluctuant resulting from the outside interference such as noise. Thus, we are interested in how the presence of noise impacts the cascade behaviors within our optimized model. Following Ref. [18], the effects of noise are introduced as an erroneous assignment of the capacity function. In detail, at a given error probability *q* (*q*_*O*_ and *q*_*I*_ for optimized and initial models, respectively), node *i*’s capacity is changed from *C*_*i*_ to $C_{i}^{'}$:

$$C_{i}^{'}=(1+\tau )C_{i}$$ (12)

where *τ*(*τ* ∈ [−0.2, 0.2]) obeys uniform distribution with zero mean. This is plausible in real-world applications due to the fact that the ignorance of the true value of the load for each node often results in an erroneous assignment of that node’s capacity. Fig 5 shows that the vulnerability of both the optimized network and the initial network increases as the error probabilities *q*_*O*_ and *q*_*I*_ increase, indicating the negative effects of noise. For the same error probability, although the redundancy capacity is reduced (please see the inset of Fig 5), the optimized network performs better, especially when *λ* is small. The optimized pattern of node capacity allocation has a stronger ability to resist noise.

## Conclusions

In summary, we propose a framework for searching for the most favorable pattern of node capacity allocation. It is modeled by a large-scale, nonlinear and multi-objective problem and solved by particle swarm optimization. We demonstrate that the optimized pattern does indeed make the network less vulnerable while at the same time reducing the cost of assigning capacities. We also introduce an indicator *H* to measure the cost-efficiency of the network. It is found that the cost-efficiency increases a lot through optimization and approximately follows the law of diminishing marginal utility. Besides, we found that the capacity-load relationship of the optimized network is nonlinear: heavily loaded nodes tend to decrease their capacity. Finally, the effect of noise is investigated, and the optimized pattern of capacity allocation shows a stronger ability to resist noise.

The optimal framework we propose here is systemic and generally applicable, which can be easily adopted in other circumstances, with the consideration of different constraints and objectives. Our results show that it is not wise to invest too much extra cost in protecting facilities when designing a network or a system due to the cost-efficiency is very small at a large maximum cost. Moreover, assigning less unoccupied capacity to the heavily loaded nodes, and more extra capacity to some lightly loaded nodes can make the network most robust as well as least cost. We believe that our work should be helpful in designing infrastructure networks from an economic point of view.
